# Woman-centered care and associated factors among midwives working in urban health centers and public and private hospitals in Tabriz, Iran: a cross-sectional study

**DOI:** 10.1186/s12978-023-01681-1

**Published:** 2023-09-12

**Authors:** Sepideh Mashayekh-Amiri, Roghaiyeh Nourizadeh, Sakineh Mohammad-Alizadeh-Charandabi, Maryam Vaezi, Shahla Meedya, Mojgan Mirghafourvand

**Affiliations:** 1 Students Research Committee, Midwifery Department, Tabriz University of Medical Sciences, Tabriz, Iran; 2 Department of Midwifery, Faculty of Nursing & Midwifery, Tabriz University of Medical Sciences, Tabriz, Iran; 3 Department of Obstetrics and Gynecology, Fellowship of Gynecology Oncology, Alzahra Teaching Hospital, Tabriz University of Medical Sciences, Tabriz, Iran; 4 South Asia Infant Feeding Research Network (SAIFRN), Faculty of Science, Medicine and Health, School of Nursing, University of Wollongong, Wollongong, Australia; 5 Social Determinants of Health Research Center, Tabriz University of Medical Sciences, Tabriz, Iran; 6 Medical Philosophy and History Research Center, Tabriz University of Medical Sciences, Tabriz, Iran

**Keywords:** Woman-Centred Care, WCCS-MSR, Midwife, Self-report, Cross-sectional study, Iran

## Abstract

**Background:**

Woman-centered care (WCC) is the cornerstone of the midwifery profession. However, no study has been conducted on WCC provided by Iranian midwives and its associated factors. Thus, this study aimed to determine WCC and factors associated with midwives’ WCC for midwives working in urban health centers and public and private hospitals in Tabriz, Iran.

**Methods:**

This cross-sectional study was the first part (i.e., the quantitative phase) of a sequential explanatory mixed-method study conducted on 575 midwives working in urban health centers and public and private hospitals in Tabriz-Iran from November 2022 to January 2023. The required data was collected by distributing a socio-demographic and job characteristics questionnaire and woman-centered care scale-midwife self-report (WCCS-MSR). To determine the factors associated with WCC, an independent t-test or one-way analysis of variance (ANOVA) was used in bivariate analysis, and a general linear model (GLM) was employed in multivariate analysis to control possible confounding variables.

**Results:**

The statistical population consisted of 575 midwives, with a response rate of 88.2%. According to the GLM, the total mean WCCS-MSR score of single [β (95% CI) 23.02 (7.94 to 38.10)] and married [β (95% CI) 21.28 (6.83 to 35.72)] midwives was significantly higher than that of divorced midwives after adjusting their demographic and job characteristics. Also, the total mean WCCS-MSR score of midwives with sufficient income was significantly higher than those with insufficient income [β (95% CI) 8.94 (0.12 to 17.77). In addition, the total mean WCCS-MSR score of midwives with < 5 years of work experience [β (95% CI) − 7.87 (− 14.79 to − 0.94)], and midwives with official-experimental employment status [β (95% CI) − 17.99 (− 30.95 to − 5.02)], was significantly lower than those with more than 5 years of work experience and contractual employment status.

**Conclusions:**

The findings indicate that marital status, level of income, years of practice, and employment status were significantly related to WCC provided by midwives. Focusing only on the midwifery community is insufficient to ensure the improved quality of WCC. However, arrangements should be made at three levels, including policy-makers, managers, and health care provider (midwives).

## Plain Language Summary

As the cornerstone of the midwifery profession, WCC represents a universal, integrated, and synonymous concept with practice, which implies focusing on women as individuals. This cross-sectional study determined WCC and its associated factors of midwives working in urban health centers and public and private hospitals in Tabriz, Iran. A sample size of 575 midwives was used to determine the factors associated with WCC. The required data was collected by distributing a socio-demographic and job characteristics questionnaire and woman-centered care scale-midwife self-report (WCCS-MSR). A generalized linear model (GLM) was used to determine the factors associated with WCC. Midwives obtained the highest total mean score in the Works in Partnership with the Woman (WP-W) subscale and the lowest total mean score in the Ensures Midwifery Philosophy Underpins Practice within the Context of the Maternity Service (EMPUP-MS) subscale in the obtainable scores, which ranged from 0 to 100. Our study found that the total mean WCCS-MSR score of single and married midwives was significantly higher than that of divorced midwives. Also, the total mean WCCS-MSR score of midwives with sufficient income was significantly higher than those with insufficient income. In addition, the total mean WCCS-MSR score of midwives with < 5 years of work experience, and midwives with official-experimental employment status, was significantly lower than those with more than 5 years of work experience and contractual employment status. The findings indicated that marital status, level of income, years of practice, and employment status were significantly related to WCC provided by midwives. Therefore, to improve the quality of WCC, identifying the mentioned factors will help policy-makers to consider facilitating measures, providing practical solutions, and designing future interventions.

## Background

Maternal mortality ratio (MMR) reduction has long been considered one of the most significant concerns of the global health [1]. According to the report published in February 23, 2023, by the World Health Organization (WHO) on behalf of the United Nations agencies, currently, every 2 min on average, a woman dies during pregnancy or childbirth [2]. Health care provided by trained midwives according to global professional standards is a primary strategy to reduce the maternal mortality ratio and improve reproductive, maternal, and infant health [3].

The care of pregnant women has been integrated into the primary health care system in Iran, and midwives have been providing high-quality maternity services in health centers for years [4]. Although midwives provide care, gynecologists are responsible for all levels of maternal care for low- and high-risk pregnant women [5]. A significant obstacle to midwives’ performance in providing intensive care services to pregnant women is the medicalization of childbirth [6]. In the biomedical model, all pregnant women are treated as at risk, the most important indicator of which is childbirth under the observation of obstetricians in advanced technology units in hospitals [7].

The inclination for early detection of complications has caused a series of preventive interventions to be carried out and to focus on “risk factors” (conditions that are not pathological but are related to it). There is often no distinction between risk factors and real-world pathology; women with “risk factors” are treated as having real-life complications [8]. In addition, Healthcare approaches currently operate according to frameworks that treat women mostly like customers. Therefore, they consider government employees responsible for the services provided [9]. In this approach, women are labeled without receiving adequate attention or having their individual needs adequately addressed [10].

While the philosophy of midwifery care is based on women’s effective participation in the self-care process, empowering and ensuring their cultural security and self-esteem have long been proposed as a humanistic concept under the title of woman-centered care (WCC). The term WCC appeared for the first time under the influence of the second-wave feminism movement in healthcare services. Despite the extensive research literature, an acceptable universal definition for WCC has not yet been offered [11]. However, it can be generally defined as “a philosophy of midwifery and a consciously chosen tool for the care management of the childbearing woman, where a collaborative relationship is formed between the woman, individual human being, and the midwife as an individual and professional through cohumanity, interaction, recognizing and respecting one another’s respective fields of expertise” [12].

According to midwives’ point of view, WCC has a three-level hierarchical framework: (1) beliefs, ethics, and personal values (intrinsic or learned) that serve as the basis for WCC [12, 13]; (2) midwives’ activity in interpersonal relationships with women and interpersonal activities of midwives with the woman’s partner, family, society, and other health professionals engaged with the woman [12, 14, 15]; (3) Achievements of midwives by providing WCC, including positive outcomes for mother and infant, self-satisfaction, and empowerment of women, as well as self-actualization as a midwife and women under her care [12, 16, 17].

WCC is associated with extensive benefits and positive outcomes for the mother and infant, especially for midwives [18]. The benefits of WCC for pregnant women include helping the physiological progress of childbirth, increasing the woman’s sense of self-sufficiency and self-confidence, facilitating the woman’s movement along with the flow of labor and delivery. For the baby, it includes strengthening the relationship between the woman and the child, making the woman more active in taking care of her child, ensuring the smooth birth of the baby, facilitating mother-baby attachment, and early initiation and continuation of breastfeeding. Also for the family, it includes the active participation of the family, strengthening the sense of security and keeping the situation under control, and ultimately seeks the satisfaction of the woman and the family. Finally, for midwives, it helps to strengthen midwives’ interaction with women, professional independence and job satisfaction of midwives [19–21].

Despite the importance of WCC, a global review of 142 documents showed that the term WCC was only identified in 3.5% of the documents, highlighting the importance and necessity of addressing this issue [22]. Although WCC is presented as the essence of midwifery care, it is a multifaceted concept based on the attitude and behavior of midwives [23]. Therefore, determining WCC and identifying associated factors will be a solid basis for designing interventions to ensure high-quality care before, during, and after childbirth. However, no study has been conducted on WCC provided by Iranian midwives and its associated factors. Thus, this study aimed to determine WCC and its associated factors of midwives working in urban health centers and public and private hospitals in Tabriz, Iran.

## Methods

As the first part (i.e., the quantitative phase) of a sequential explanatory mixed-method study [24], this cross-sectional study was conducted on 575 midwives working in urban health centers and public and private hospitals in Tabriz from November 2022 to January 2023.

### Participants and sampling

In this study, the required data were collected using the census sampling method from all urban health centers (n = 92), public hospitals (e.g., Alzahra, Taleghani, Imam Ali, and Alghadir), and private hospitals (e.g., Shahriar, Shams, Behboud, and Valiasr International Hospital) in Tabriz-Iran. Inclusion criteria were midwives working in urban health centers, public and private hospitals in Tabriz, living in Tabriz, holding at least a Bachelor’s degree, and having work experience of at least 6 months. Exclusion criteria were midwives with a history of depression, use of antidepressants including tricyclic antidepressants (TCAs), selective serotonin reuptake inhibitors (SSRIs), monoamine oxidase inhibitors (MAOIs), and noradrenaline (NA) and also a history of traumatic events, such as divorce, death of first-degree relatives (FDRs), and diagnosis of terminal or refractory illness for a first degree relatives during the last 3 months.

At first, the researcher gave a comprehensive explanation of the goals and methodology to all qualified midwives. Then, written informed consent was obtained from midwives willing to participate in the study. In addition, the socio-demographic and job characteristics questionnaire and Woman-Centred Care Scale-Midwife Self Report (WCCS-MSR) were completed anonymously.

### Measures

Two questionnaires were used in the present study: (1) a socio-demographic and job characteristics questionnaire and (2) WCCS-MSR.

#### Socio-demographic and job characteristics questionnaire

The socio-demographic and job characteristics questionnaire includes information such as age, spouse’s age, marriage age, marital status, spouse’s job, level of income, level of education, workplace, employment status, years of practice, hours worked per week, and getting a physiological childbirth workshop certificate.

#### Woman-centred care scale-midwife self report (WCCS-MSR)

WCCS-MSR was applied to evaluate WCC by midwives. It was first designed by Davis et al. [25]. It included five domains [Meets the Unique Needs of the Woman (MUN_W, 12 items), Balances the Woman’s Needs within the Context of the Maternity Service (BWN_MS, 5 items), Ensures Midwifery Philosophy Underpins Practice within the Context of the Maternity Service (EMPUP_MS, 4 items), Working Collaboratively for Evidence-Based Practice (WC_EBP, 7 items) and Works in Partnership with the Woman (WP_W, 12 items)]. This is a 40-item questionnaire on a 7-point Likert scale [very untrue of me (1) and very true of me (7)]. The minimum and maximum scores in this questionnaire are 40 and 280, respectively. Higher scores indicate higher-quality WCC by midwives. The validity and reliability of the questionnaire have been demonstrated in midwifery personnel in Australia. The internal consistency of the questionnaire was reported to be 0.92 using Cronbach’s alpha coefficient [25]. Also, in the present study, the reliability of the tool was confirmed as 0.94 with Cronbach’s alpha and McDonald’s omega coefficients and 0.98 with the intraclass correlation coefficient (ICC).

### Statistical analysis

The collected data were analyzed by SPSS (Version 25.0) (IBM Corp, Armonk, NY, USA). In descriptive statistics, categorical variables were described based on frequency and percentage, and continuous variables were defined based on mean and standard deviation (SD). Quantitative data normality was determined using visual inspection, skewness, and kurtosis, implying normal data distribution. The relationship between midwives’ socio-demographic and job characteristics and the ‘WCCS-MSR score’ variable was explored using an independent t-test and one-way ANOVA. In the next step, statistically significant variables (P < 0.1) were imported into the general linear model (GLM) to determine the effect of independent variables on the dependent variable (i.e., WCC) (P < 0.05 means statistically significant).

### Ethical considerations

The present study was approved by the Ethics Committee of Tabriz University of Medical Sciences (Ethical approval code: IR.TBZMED.REC.1401.051; date: 6, April 2022). Written informed consent was obtained from all participants. Before data collection, administrative permission was obtained from hospital chief executive officers (CEOs) and physicians-in-charge in health centers. The anonymous survey coding method was used to preserve the midwives’ privacy. Moreover, a comprehensive explanation was given to the midwives about the research process and objectives, confidentiality, and freedom to withdraw their participation, at each step in the research process.

## Results

### Socio-demographic characteristics of the midwives

Five hundred seventy-five midwives participated in this study, with a response rate of 88.2%. The mean (SD) age of midwives was 38.0 (7.9) years, with a minimum age of 23 years and a maximum age of 64 years (38.8% of midwives were in the age range of 30–40 years). Likewise, the mean (SD) age of midwives’ spouses was 42.9 (8.5) years, with a minimum age of 21 and a maximum age of 71. Midwives’ average age of marriage was 25.6 (4.4) years. Most participants [i.e., 430 (74.8%)] were married. About half of the midwives (49.6%) reported that their income level was relatively sufficient. Besides, about 40% of midwives’ spouses were employees (Table 1).

**Table 1:** Socio-demographic and job characteristics among midwives (n = 575)

Variables	n (%)
Age (year)^a^	38.0 (7.9)
Spouse’s age (year)^a^	42.9 (8.5)
Married age (year)^a^	25.6 (4.4)
Marital status	
Single	131 (22.8)
Married	430 (74.8)
Divorced	14 (2.4)
Spouse’s job	
Employee	230 (40.0)
Specialists-managers	44 (7.7)
Self-employment	148 (25.7)
Income	
Completely sufficient	44 (7.7)
Somewhat sufficient	285 (49.6)
Insufficient	246 (42.8)
Educational level	
BSc	520 (90.4)
Postgraduate (MSc, PhD)	55 (9.6)
Employment sector	
Public	130 (22.6)
Private	95 (16.5)
Health center	350 (60.9)
Employment status	
Compulsory service program	41 (7.1)
Official-experimental	18 (3.1)
Official-definitive	248 (43.1)
Contractual	268 (46.6)
Years of practice	
> 5 years	109 (19.0)
≥ 5 years	466 (81.0)
Hours worked per week	
18 to 36 h	55 (9.5)
37 to 48 h	390 (67.8)
< 48 h	130 (22.6)
Physiological birth certificate	
Yes	227 (39.5)
No	348 (60.5)

*MSc* Master of Science, *PhD* Philosophiae doctor

^a^Mean ± SD

### Job characteristics of the midwives

As for the workplace, more than half of the midwives (60.9%) worked in health centers. Most (46.6%) had contractual employment status (were contractually employed). Most of them (90.4%) had a bachelor’s degree. Also, about 19% were junior midwives, while 81% were senior midwives. The majority had 5 or more years of work experience. More than half (60.5%) did not have a certificate of participation in physiological childbirth classes. A summary of other socio-demographic and job characteristics of midwives is presented in Table 1.

### Woman-centred care scale-midwife self report (WCCS-MSR) and sub‑scales

**Fig. 1:**
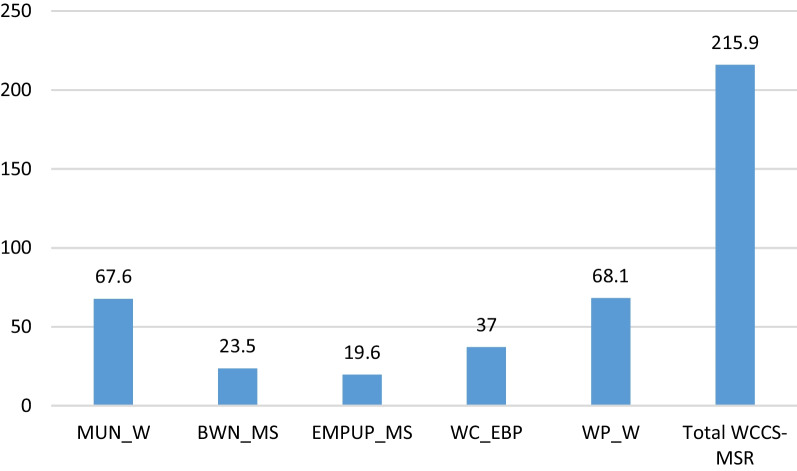
Distribution of percentage mean score of WCCS-MSR scale and its subscales among midwives working in the public and private hospitals in Tabriz, Iran

In the present study, the total mean (SD) of the WCCS-MSR score was 215.90 (27.44) in the range of obtainable scores from 40 to 280. Midwives obtained the highest total mean score in the WP-W subscale [68.14 (10.69)] and the lowest total mean score in the EMPUP-MS subscale [19.64 (3.72)] in the obtainable scores range from 0 to 100 (Fig. 1; Table 2).

**Table 2:** Status of woman-centered care and its subscales in midwives (n = 575)

Variables	Mean (SD^a^)	Obtainable score range	Obtained score range
Total score of WCCS-MSR	215.90 (27.44)	0–100	4.2–100
MUN-W	67.61 (9.82)	0–100	2.8–100
BWN-MS	23.50 (4.63)	0–100	0–100
EMPUP-MS	19.64 (3.72)	0-100	0–100
WC-EBP	37.01 (6.09)	0–100	0–100
WP-W	68.14 (10.69)	0–100	0–100

*WCC-MSR* woman-centered care-midwife self-report, *MUN-W* meets the Unique Needs of the Woman, *BWN-MS* balances the Woman’s Needs within the Context of the Maternity Service, *EMPUP-MS* Ensures Midwifery Philosophy Underpins Practice within the Context of the Maternity Service, *WC-EBP* Working Collaboratively for Evidence-Based Practice, *WP-W* works in partnership with the woman

^a^Standard deviation

### Factors associated with woman-centered care among midwives

The results of bivariate tests (one-way ANOVA and independent t-test) indicated a significant statistical relationship between the total WCCS-MSR score and marital status (P = 0.008), employment status (P = 0.020), and years of practice (P = 0.038) (Table 3). Then, variables associated with WCC with P < 0.1 and WCCS-MSR score were imported into the GLM as independent and dependent variables, respectively. According to the adjusted general linear model results, marital status, level of income, years of practice, and employment status were significantly related to the WCCS-MSR score. By controlling the effect of all other variables in the model, single [β (95% CI) 23.02 (7.94 to 38.10); p = 0.003] and married [β (95% CI) 21.28 (6.83 to 35.72); p = 0.004] midwives obtained a significantly higher WCCS-MSR score than their divorced midwives. Midwives with sufficient income showed a significantly higher level of WCC than those with insufficient income [β (95% CI) 8.94 (0.12 to 17.77); p < 0.05].

**Table 3:** The relationship of socio-demographic characteristics with women-centered care in midwives based on bivariate analysis (n = 575)

Variables	N	Mean ± SD	P-value
Age (year)			
> 30	101	212.75 ± 24.60	0.169^b^
≤ 30	474	216.57 ± 27.98	
Marital status			
Single	131	216.89 ± 22.79	0.008^a^
Married	430	216.33 ± 27.20	
Divorced	14	193.50 ± 55.39	
Spouse’s job			
Employee	230	216.43 ± 28.12	0.758^a^
Specialists-managers	44	218.43 ± 20.42	
Self-employment	148	215.10 ± 27.46	
Income			
Completely sufficient	44	223.68 ± 21.01	0.059^a^
Somewhat sufficient	285	216.75 ± 25.39	
Insufficient	246	213.53 ± 30.36	
House type			
Personal	485	216.65 ± 27.28	0.141^b^
Rental	90	211.88 ± 28.06	
Education			
BSc	520	215.97 ± 27.74	0.840^b^
Postgraduate (MSc, PhD)	55	215.25 ± 24.54	
Employment status^c^			
Compulsory service program	41	213.44 ± 23.58	0.020^a^
Official-experimental	18	196.72 ± 45.81	
Official-definitive	248	216.23 ± 24.28	
Contractual	268	217.26 ± 28.78	
Employment sector			
Health center	350	216.04 ± 28.50	0.972^a^
Public hospital	130	215.97 ± 22.71	
Private hospital	95	215.29 ± 29.51	
Years of practice			
> 5	109	211.00 ± 30.53	0.038^b^
≤ 5	466	217.05 ± 26.56	
Hours worked per week			
18 to 36 h	55	214.20 ± 25.81	0.890^a^
37 to 48 h	390	216.08 ± 27.59	
> 48 h	130	216.07 ± 27.80	
Physiological birth certificate			
Yes	227	216.24 ± 26.86	0.808^b^
No	348	215.68 ± 27.84	

*MSc* Master of Science, *PhD* Philosophiae doctor

^a^One-way ANOVA

^b^Independent samples t test

^c^ Employment status: official employment status is classified into two categories: official-experimental and official-definitive. Midwives work on a trial basis before obtaining official-definitive employment status. If they are qualified, their employment status changes from official-experimental to official-definitive employment status

On the other hand, midwives with < 5 years of work experience exhibited a significantly lower level of WCC compared to midwives with work experience equal to or more than 5 years [β (95% CI) − 7.87 (− 14.79 to − 0.94); p < 0.05]. Likewise, midwives with official-experimental employment status obtained a significantly lower WCCS-MSR score than midwives with contractual employment status [β (95% CI) − 17.99 (− 30.95 to − 5.02); p = 0.007]. A non-significant decrease was also reported in the WCCS-MSR score of midwives with official-definitive employment status [β (95% CI) − 1.69 (− 6.46 to 3.09); p = 0.489]. Also, compulsory service program midwives obtained a non-significantly higher WCCS-MSR score than contractually employed midwives [β (95% CI) 1.62 (− 8.99 to 12.23); p = 0.765] (Table 4).

**Table 4:** The relationship of sociodemographic characteristics with WCC in midwives based on the General Linear Model (n = 575)

Characteristics	Unadjusted^a^		Adjusted	
	β (95% CI^b^)	P	β (95% CI^b^)	P
Marital status (reference: divorced)				
Single	23.39 (8.34 to 38.44)	0.002	23.02 (7.94 to 38.10)	0.003
Married	22.83 (8.29 to 37.37)	0.002	21.28 (6.83 to 35.72)	0.004
Income adequacy (reference: not enough at all)				
Quit enough	10.15 (1.36 to 18.95)	0.024	8.94 (0.12 to 17.77)	0.047
Relatively enough	3.22 (− 1.46 to 7.89)	0.177	2.99 (− 1.74 to 7.73)	0.214
Years of practice (reference: ≥ 5)				
< 5	− 6.04 (− 11.76 to − 0.33)	0.038	− 7.87 (− 14.79 to − 0.94)	0.026
Employment status (reference: contractual)				
Compulsory service program	− 3.82 (− 12.80 to 5.16)	0.404	1.62 (− 8.99 to 12.23)	0.765
Official-experimental	− 20.54 (− 33.60 to − 7.50)	0.002	− 17.99 (− 30.95 to − 5.02)	0.007
Official- definitive	− 1.03 (− 5.75 to 3.69)	0.668	− 1.69 (− 6.46 to 3.09)	0.489

^a^Significant variables with p < 0.1 in the bivariate analysis were included in a multivariate analysis

^b^95% confidence interval

## Discussion

As the cornerstone of the midwifery profession, WCC represents a universal, integrated, and synonymous concept with practice, which implies focusing on women as individuals [26]. Despite the relevant qualitative research and many recommendations regarding the provision of WCC by midwives, as long as the pertinent and involved factors are not identified by conducting quantitative research, desired results will not be produced to improve the status and reflect the results in clinical medicine. Identifying midwives’ socio-demographic factors can help improve organizations’ performance, planning, and healthcare procedures [27]. Therefore, to our knowledge, the present study has been conducted for the first time to investigate WCC and associated factors regarding midwives working in urban health centers and private and public hospitals in Tabriz, Iran. The results demonstrated that midwives’ socio-demographic and job characteristics, such as marital status, level of income, years of practice, and employment status, affected their WCCS-MSR score.

The mean (SD) of the WCCS-MSR score was calculated to be 215.90 (27.44) in the present study. Midwives obtained the highest total mean score in the WP-W subscale [68.14 (10.69)] and the lowest total mean score in the EMPUP-MS subscale [19.64 (3.72)] in the obtainable scores range from 0 to 100. In Davis et al.’s [25] study, the total mean WCCS-MSR score was 237.93 (38.78). Also, similar to our research, midwives obtained the highest [77.61 (7.91)] and lowest [17.43 (6.69] scores in WP-W and EMPUP-MS subscales.

Factors associated with WCC identified in the present study are consistent with the results of some relevant studies. Consistent with our research, in Sattarzadeh et al.’s [28] study entitled “Determining socio-demographic predictors of midwives’ practice and knowledge regarding preconception care,” age, level of education, employment status, and work experience were predictors of midwives’ knowledge. In contrast, age, employment status, and professional liability were predictors of midwives’ practice. On the other hand, inconsistent with our study, Ogbuabor et al. [29] conducted a mixed-method study to explore midwives’ perspectives on person-centered maternity care (PCMC) in public hospitals in southeastern Nigeria. The results indicated that midwives’ perception of PCMC was not significantly related to socio-demographic factors. This difference can probably be attributed to the two studies’ different methodologies. In Ogbuabor et al.’ [29] study, only four public hospitals were considered. In contrast, the present study used the census sampling method by considering all urban health centers and public and private hospitals.

The first factor identified in this study was the midwives’ marital status. According to the results of the present study, single and married midwives obtained a higher WCCS-MSR score than their divorced midwives. This can probably be attributed to the fact that women have to grapple with more changes and challenges compared to men after divorce [30], including economic problems, depression, reduced level of life satisfaction, playing various roles, impaired social interactions, child custody problems, callousness, and declining mental and physical health [31, 32]. Therefore, divorced midwives seem to be under more pressure and stress as they face the above problems and professional issues, which negatively affects their WCCS-MSR score. In this respect, Khakbazan et al. [33] conducted a qualitative content analysis to identify the factors involved in providing high-quality midwifery care. They introduced two categories of influential factors: (1) individual factors (including personal efficiency, staff’s psychological status, value-centeredness, cultural-educational issues, and professional attachment) and (2) professional-organizational factors (professional characteristics, patient characteristics, personal-professional welfare, and professional empowerment system, value-centered culture of organization, and organizational monitoring and evaluation). As a result, individual factors, including staff’s psychological status, affected their maternity care level and quality.

The second identified factor was the midwives’ level of income. In this study, midwives with sufficient income levels showed a higher maternity care level than those with insufficient income. A possible explanation could be that income is positively related to health-related quality of life. As a result, midwives with higher quality of life have more motivation to provide WCC. In this regard, Khosravi et al.’s [34] study on the strategies to improve the quality of midwifery care in Iran through the analysis of midwifery specialists’ perspectives revealed that midwives’ encouragement and motivation was the most discussed topic by most of the participants. Accordingly, [timely] payment of midwives’ remuneration was one of the most critical topics addressed by the participants. They believed that midwives would not have enough motivation and desire to provide high-quality services if midwifery services were not well compensated. Previous studies have also addressed the importance of midwives’ adequate income to provide the best services by these health care providers. They believed most midwives were dissatisfied with their salaries in hospitals and health centers [34, 35]. Furthermore, Ogbuabor et al.’s [29] study showed that working conditions and systemic factors could limit the scope of maternity care, meaning midwives must work long hours under challenging conditions. In addition, several studies indicated poor motivation of midwives due to poor salaries, lack of support from doctors, and negative hierarchical relationships with doctors [35–37].

Another factor identified was years of practice. The results of the current study demonstrated that midwives with < 5 years of work experience exhibited a significantly lower level of WCC compared to midwives with work experience equal to or more than 5 years. A possible explanation for this difference could be that midwives with less work experience have less knowledge and experience compared to midwives with more work experience, resulting in lower scores in WCC scale. In this regard, in their study entitled “Iranian Midwives’ Awareness and Performance of Respectful Maternity Care during Labor and Childbirth,” Haghdoost et al. [38] suggested that work experience with a Master’s degree in midwifery had a significant positive effect on awareness and performance of respectful maternity care (RMC). In this regard, an increase of 1 year of practice was associated with a 0.968 increase in midwives’ awareness of RMC and a 1.18% unit increase in their performance of RMC. Similarly, compared to midwives with an Associate’s degree in midwifery education, midwives with a Master’s degree in midwifery education showed a 2.41% increase in awareness of RMC and a 0.59% increase in performance of RMC. Although the results of years of practice were consistent with those of our study, the present study did not observe any relationship between the level of education and the provision of WCC, which can probably be attributed to the fact that postgraduate midwives [Doctor of Philosophy (PhD) and Master of Science degrees] constituted a small part of our sample size, and the majority of them had a bachelor’s degree. Also, our study aimed to identify factors associated with WCC. In contrast, the above research addressed RMC, which was compared due to the lack of similar studies on WCC.

Finally, the last factor identified in the present study was midwives’ employment status. The present study revealed that midwives with official-experimental employment status obtained a lower WCCS-MSR score than those with contractual employment status. This can probably be attributed to the fact that while contractual midwives provide better quality care due to the uncertainty in employment status and job insecurity, their official counterparts obtained a lower WCCS-MSR score because they are almost confident of their job security [39]. On the other side, official-definitive midwives obtained an insignificantly lower WCCS-MSR score than contractual midwives. This can probably be attributed to the fact that their performance was unconsciously positively affected by their years of practice and, at the same time, job security and higher years of practice. Thus, they obtained a higher WCCS-MSR score than official-experimental midwives and provided higher-quality healthcare services.

In this respect, Khosravi et al.’s [34] study showed that the participants’ most critical human resource (HR) strategies were mainly focused on employment status and increasing the midwifery workforce. The head of the midwifery association pointed out that according to the law approved by the Ministry of Health, there must be 12 midwives for every 1000 live births in hospitals, but at present, only 8–9 midwives are available for every 1000 live births in Iranian hospitals. Of the 8000 employed midwives, 2700 are provisionally employed, and after 2 years, most of them become unemployed. As for midwives’ employment status, the need for midwifery care and services is on the rise [34], and more midwives should be employed, considering the implementation of the urban family doctor plan in Iran and that vaginal delivery is free of charge in governmental hospitals [40].

### Strengths and limitations

To the best of our knowledge, the present study, for the first time, investigated WCC and associated factors for Iranian midwives. Therefore, this study will serve as a sound basis for designing interventions by discovering factors associated with WCC. Among other strengths of the current study are the use of the census sampling method and the evaluation of WCC provided by midwives in urban health centers and public and private hospitals.

However, the present study also had limitations. This cross-sectional study could not inspect the causal relationship between different variables and WCC, creating the need for more prospective studies in this field. In addition, midwives may have social desirability bias because they may refuse to honestly reflect their genuine care status so that their work performance is not undermined. The results of the study may also be affected because sampling was not performed in rural health centers (clinics) and on midwives working in practices. Finally, the results reflect only the situation in Iran. Since different countries implement healthcare policies differently, the conclusions may not be generalizable to other countries. On the other hand, despite the relevant qualitative research, little relevant quantitative research was found to compare the results obtained in the present study with those of other related studies. Thus, conducting more quantitative studies worldwide to identify factors affecting the quality of WCC provided by midwives in hospitals and health centers is recommended.

## Conclusion

The findings indicated that marital status, level of income, years of practice, and employment status were significantly correlated with WCC provided by midwives. Focusing only on the midwifery community is insufficient to ensure the improved quality of WCC. However, arrangements should be made at three levels, including policy-makers, managers, and health care provider (midwives). At the first level, policymakers should design and implement national WCC guidelines. On the second level, hospital managers should take a series of targeted measures, including holding educational workshops and programs for midwives on the notion of WCC (using the skilled workforce to train less experienced workforce and sharing experience), allocating sufficient workforce to reduce workload and subsequently increase care quality (especially in the night shift due to higher delivery rates), providing a friendly workplace, using midwifery models based on WCC in clinical environments, offering material and spiritual incentives to midwives, using measures to reduce conflict of interests between midwifery groups and women, encouraging teamwork and cooperation, and finally, getting feedback from women following the care provided to them, to improve the quality of current WCC. Finally, midwives should offer WCC to all women during preconception, pregnancy, intrapartum, and postpartum periods regardless of their socio-economic status. Given the fifth sustainable development goals (SDGs), which is empowering women and girls, providing WCC by midwives and conducting future longitudinal research will play a crucial role in achieving this goal.

## Data Availability

The datasets used and/or analyzed during the current study are available from the corresponding author upon reasonable request.

## Abbreviations

WCC: Woman-Centred Care
WCCS-MSR: Woman-Centred Care Scale-Midwife Self Report
MUN_W: Meets the unique needs of the woman
BWN_MS: Balances the woman’s needs within the context of the maternity service
EMPUP_MS: Ensures midwifery philosophy underpins practice within the context of the maternity service
WC_EBP: Working collaboratively for evidence-based practice
WP_W: Works in partnership with the woman
MMR: Maternal mortality rate
WHO: World health organization
PHC: Primary health care
SPSS: Statistical Package for Social Science
ANOVA: One-way analysis of variance
GLM: Generalised linear model
SD: Standard deviation
CI: Confidence interval
RMC: Respectful maternity care
PCMC: Person-Centered Maternity Care
SDG: Sustainable development goals
